# Healthy Serum-Derived Exosomes Improve Neurological Outcomes and Protect Blood–Brain Barrier by Inhibiting Endothelial Cell Apoptosis and Reversing Autophagy-Mediated Tight Junction Protein Reduction in Rat Stroke Model

**DOI:** 10.3389/fncel.2022.841544

**Published:** 2022-03-03

**Authors:** Lin-Yan Huang, Jin-Xiu Song, Heng Cai, Pei-Pei Wang, Qi-Long Yin, Yi-De Zhang, Jie Chen, Ming Li, Jia-Jia Song, Yan-Ling Wang, Lan Luo, Wan Wang, Su-Hua Qi

**Affiliations:** ^1^Xuzhou Key Laboratory of Laboratory Diagnostics, School of Medical Technology, Xuzhou Medical University, Xuzhou, China; ^2^Pharmacology College, Xuzhou Medical University, Xuzhou, China

**Keywords:** serum exosomes, stroke, BBB, apoptosis, autophagy

## Abstract

Blood–brain barrier (BBB) dysfunction causing edema and hemorrhagic transformation is one of the pathophysiological characteristics of stroke. Protection of BBB integrity has shown great potential in improving stroke outcome. Here, we assessed the efficacy of exosomes extracted from healthy rat serum in protection against ischemic stroke *in vivo* and *in vitro*. Exosomes were isolated by gradient centrifugation and ultracentrifugation and exosomes were characterized by transmission electron microscopy (TEM) and nanoparticle tracking video microscope. Exosomes were applied to middle cerebral artery occlusion (MCAO) rats or brain microvascular endothelial cell line (bEnd.3) subjected to oxygen-glucose deprivation (OGD) injury. Serum-derived exosomes were injected intravenously into adult male rats 2 h after transient MCAO. Infarct volume and gross cognitive function were assessed 24 h after reperfusion. Poststroke rats treated with serum-derived exosomes exhibited significantly reduced infarct volumes and enhanced neurological function. Apoptosis was assessed via terminal deoxynucleotidyl transferase (TdT)-mediated dUTP nick-end labeling (TUNEL) staining and the expression of B-cell lymphoma-2 (Bcl-2), Bax, and cleaved caspase-3 24 h after injury. Our data showed that serum exosomes treatment strikingly decreased TUNEL^+^ cells in the striatum, enhanced the ratio of Bcl-2 to Bax, and inhibited cleaved caspase-3 production in MCAO rats and OGD/reoxygenation insulted bEnd.3 cells. Under the consistent treatment, the expression of microtubule-associated protein 1 light chain 3B-II (LC3B-II), LC3B-I, and Sequestosome-1 (SQSTM1)/p62 was detected by Western blotting. Autolysosomes were observed via TEM. We found that serum exosomes reversed the ratio of LC3B-II to LC3B-I, prevented SQSTM1/p62 degradation, autolysosome formation, and autophagic flux. Together, these results indicated that exosomes isolated from healthy serum provided neuroprotection against experimental stroke partially via inhibition of endothelial cell apoptosis and autophagy-mediated BBB breakdown. Intravenous serum-derived exosome treatment may, therefore, provide a novel clinical therapeutic strategy for ischemic stroke.

## Introduction

Stroke is one of the leading causes of morbidity and mortality worldwide (Benjamin et al., 2017) and ranks the first lethal cause in China (Yang et al., 2013; Gao et al., 2018), with ischemic stroke accounting for about 87% of total occurrence (Benjamin et al., 2017). One of the pathophysiological characteristics of ischemic stroke is the destruction of the blood–brain barrier (BBB), which significantly promotes the progression of vasogenic edema formation and hemorrhagic transformation (Mracsko and Veltkamp, 2014; Alluri et al., 2015; Turner and Sharp, 2016). Various reports have indicated that preventing BBB dysfunction improves functional outcome after ischemic stroke (Reeson et al., 2015; Jiang et al., 2018; Balkaya et al., 2021). Inhibition of matrix metalloproteinase-9 (MMP-9) activity also contributed to delayed thrombolysis-induced hemorrhagic transformation (Chen H.S. et al., 2015; Chen et al., 2019). Studies with plasma or blood transfusion showed great potential in treating stroke by protecting BBB integrity (Ren et al., 2020; Mamtilahun et al., 2021). Therefore, it is urgent to develop effective therapeutic strategies to prevent the BBB dysfunction in ischemic stroke.

The BBB consists of brain microvascular endothelial cells (BMECs), astrocytes, pericytes, neurons, and extracellular matrix around the vessels, which contains type IV collagen, fibronectin, laminin, heparan sulfate, and perlecan (Yang and Rosenberg, 2011a; Thomsen et al., 2017; Sacks et al., 2018; Page et al., 2020). BMECs are the scaffold of the BBB and their death results in catastrophic failure of BBBs integrity (ElAli et al., 2011; Wang et al., 2011). Necrosis and apoptosis are the two main forms of cell death after stroke and sequential activation of necroptosis and apoptosis synergistically mediates vascular destruction and neuronal injury in stroke (Yong et al., 2019; Naito et al., 2020). Pericyte apoptosis also increases BBB permeability, which, in turn, diminishes the stability of the brain microenvironment (Armulik et al., 2010; Liu S. et al., 2012). The biochemical compounds targeting endothelial cell and pericyte apoptosis exert promising effects on functional recovery after central nervous system injury (Hu et al., 2017; Song et al., 2018; Wu et al., 2020).

The biochemical features of the BBB damage include decreased expression of tight junction component proteins such as zonula occludens (ZOs), claudins, and occludin as well as the regulation of the functional expression of endogenous BBB transport proteins such as ATP-binding cassette transporters and solute carrier transporters (Abdullahi et al., 2018). Previous studies pointed out that MMPs activation was involved in the destruction of the BBB after ischemic stroke (Bauer et al., 2010; Kumari et al., 2011; Zhang et al., 2018). This included MMP-2 activated by hypoxia-inducible factor-1α (HIF-1α) and MMP-3 and MMP-9 triggered by proinflammatory cytokines (Yang and Rosenberg, 2011a). MMP-2 and MMP-9 directly destroyed the BBB by degrading the constituent proteins of tight junction (Liu J. et al., 2012; Reuter et al., 2015; Qi et al., 2016). Besides, autophagy was another degradation pathway that clears damaged or unnecessary intracellular proteins. Autophagy-lysosome-mediated degradation of occludin and ZO-1 protein also contributed to the BBB disruption (Zhang et al., 2018; Kim et al., 2020; Yang et al., 2021).

Exosomes are extracellular vesicles (30–130 nm in diameter) released by most cell types after the fusion of multivesicular bodies with the plasma membrane (Colombo et al., 2014). Exosomes exist in the circulation and contain a variety of proteins, nucleic acids, and lipids from host cells, which promote cell-to-cell communication and regulate receptor cell functions (Robbins et al., 2016). Exosomes can cross the BBB and can be transported into brain through pinocytosis (Colombo et al., 2014). Recent study using exosomes to treat ischemic stroke attributes to their cargo, which include DNA, RNA, microRNA, proteins, and lipids (Chen and Chopp, 2018). Exosomes have shown promising results in ischemic stroke either by their intrinsic therapeutic characteristics, which can result in angiogenesis and neurogenesis or by acting as competent, biocompatible drug delivery vehicles to transport neurotherapeutic agents into the brain (Nozohouri et al., 2020).

Exosomes from mesenchymal stem cells, neural stem cells, astrocytes, and microglia have shown great potential in functional recovery of ischemic stroke (Doeppner et al., 2015; Pei et al., 2019; Song et al., 2019; Zhang et al., 2020). Considering the single source of the exosomes from single cellular type, plasma (or serum) contains exosomes from all the cellular origins and this makes plasma exosomes have a comprehensive therapeutic effect (Bei et al., 2017; Kang et al., 2019). The yield of plasma (or serum) exosomes was largely higher than the exosomes secreted by cells, for example, circulating reticulocytes produce larger amount of exosomes (∼200 μg per day) than dendritic cells (∼1–2 μg per 10^6^ dendritic cells per day) (Blanc et al., 2005). Also, it has been reported that plasma exosome subjected to ischemic stimuli exerted neuroprotection through HIF-1α signals (Li et al., 2019). Circulating plasma exosomes deliver HSP70 protein to modulate redox oxygenic species to suppress neuron cell apoptosis and the BBB damage against cerebral ischemia/reperfusion injury (Jiang et al., 2020). In this study, we confirmed the effect of serum exosomes derived from healthy donor on the neurological function and the BBB integrity of the rats subjected to ischemic/reperfusion injury. Most importantly, we figured out the underlying mechanism of serum exosomes on the BBB integrity through assessing the effect of serum exosomes on the apoptosis and autophagy.

## Materials and Methods

### Animals

Male Sprague-Dawley rats (230–250 g) were provided by the Animal Experiment Center of Xuzhou Medical University. The animals were housed in 12 h light/dark cycle conditions with controlled temperature and humidity and were supplied adequate food and water before the experiment. The animal experiment was conducted in accordance with the national and institutional guidelines on ethics and biosafety and has been conducted after the approval of the Local Animal Care Committees (License ID: 201907W079). All the animal experiments were approved and regulated by the Committee on the Use of Live Animals in Teaching and Research of Xuzhou Medical University.

### Isolation and Characterization of Serum Exosomes

In total, 30 rats were used to isolate serum exosomes for the whole study. About 7ml whole blood could be collected from one sham rat via the abdominal aorta into a vacutainer. The whole blood was allowed to clot at room temperature for 30 min. The serum (1–2 ml) was obtained by centrifugation at 2,500 g for 10 min. The serum was ultracentrifuged at 100,000 g for 60 min (Beckman Optima L-100XP, Beckman, United States) and the pellets were resuspended in phosphate-buffered saline (PBS) and then ultracentrifuged at 150,000 g for another 90 min. The pellets were resuspended in 1 ml PBS and passed through the 0.22 μm filter (Millipore, SLGPR33RB) to obtain sterile exosomes. The protein concentration of exosomes was determined with bicinchoninic acid (BCA) commercial kit (Vazyme, China) as manufacturer’s instruction. The protein concentration of 1 ml exosomes is 0.1–0.2 mg/μl. The exosomes were aliquoted and stored at –80°C until use.

The serum exosomes (labeled as Con-exo) were observed using transmission electron microscopy (TEM) to identify the morphology. The size and concentration distribution profile of serum exosomes were analyzed using a Multiple-Laser ZetaView^®^ f-NTA Nanoparticle Tracking Analyzers (Particle Metrix, Germany). The exosome surface markers were confirmed by Western blotting using antibodies against CD9 and TSG101.

### Middle Cerebral Artery Occlusion Model

Focal cerebral ischemia was induced by the intraluminal suture MCAO method (Longa et al., 1989; Gong et al., 2015). Briefly, after anesthesia with inhalation of 4% isoflurane, the left common carotid artery (CCA), internal carotid artery (ICA), and external carotid artery (ECA) were exposed through a midline incision of the neck. A 3–10 silica gel coated with nylon suture was used as an embolus and inserted to the origin of MCA via the ECA to block MCA for 2 h and then the suture was withdrawn for 24 h reperfusion. In the sham group, the suture was inserted 5 mm from the incision and no cerebral ischemia was induced. After the operation, animals were transferred into an intensive care chamber with the maintained temperature at 37°C.

### Blood–Brain Barrier Penetration Test

The penetration ability of Con-exo crossing the BBB was evaluated using 1,1′-dioctadecyl-3,3,3′,3′-tetramethylindocarbocyanine perchlorate (Dil) labeled Con-exo. The label of Con-exo with Dil was performed, as previously described (Elashiry et al., 2021). Briefly, Con-exo was incubated with Dil (C1036, Beyotime, China) at 37°C in the dark for 15 min. After centrifugation at 100,000 g for 60 min, the pellet was resuspended and washed with PBS twice. Then, Dil-labeled Con-exo was intravenously injected to sham and MCAO rats. The rats were sacrificed after injection for 0.5, 6, and 24 h, respectively. The rats were perfused with 4% paraformaldehyde (PFA) by cardiac transfusion. Then, the brains were collected and fixed in 4% PFA. After dehydration with 30% sucrose solution, the brains were embedded with Tissue-Tek^®^ OCT Compound and prepared for cryosection. After cryosection, the slides were captured under fluorescence microscope (Olympus BX51, Japan). The fluorescence intensity of Dil was analyzed with Image J software (Maryland, United States).

### Experimental Designs and Serum Exosome Administration

For 2,3,5-triphenyltetrazolium chloride (TTC) staining experiments, 25 rats were totally used. Serum exosomes (400, 800, and 1,600 μg/kg) were injected intravenously after ischemia for 2 h, followed with reperfusion for 24 h. The optimal dosage of exosomes (800 μg/kg) was chosen from TTC staining of rat focal ischemia/reperfusion injury model for the subsequent experiments. Then, 21 rats were randomly divided into the three groups: sham (labeled as control, *n* = 5), MCAO 2 h/reperfusion 24 h injected with equal volume of PBS (labeled as MCAO, *n* = 8), and MCAO 2 h/reperfusion 24 h plus 800 μg/kg exosomes (labeled as Con-exo, *n* = 8). The MCAO rats were only subjected to Con-exo injection once during the experiment.

### Infarct Volume Measurement

Infarct volume was determined by TTC (A610558, Shanghai Sangon, China) staining. After 2 h of ischemia and reperfusion for 24 h, the animals were sacrificed and the brains were quickly dissected. The brains were placed at –20°C for 30 min. Then, the brain was cut into 2 mm coronal sections and immersed in 2% TTC solution for 30 min at 37°C. The sections were preserved in 4% PFA solution overnight and then photographed. Infarct volume in the hemispheric lesion area was calculated by summation of unstained areas of all the slices and multiplied by the slice thickness (2 mm) and quantitatively analyzed with Image J software (Image J software, Maryland, United States). Relative infarct volume percentage was calculated with following formula: (uninfarct hemisphere area-infarct hemisphere uninfarct area)/uninfarct hemisphere area × 100.

### Neurobehavioral Test

The Modified Neurological Severity Score (mNSS) was assessed to evaluate the animal neurological deficit according to previous study (Chen et al., 2001). Neurological function was graded on the scales from 0 to 18 (normal score, 0; maximal deficit score, 18). The mNSS includes motor, sensory, reflex, and balance tests. The mNSS details were shown in Supplementary File. Tests were carried out by the researchers who were blind to the experimental design.

### Open Field Test

Rat spontaneous locomotor activity was measured using an open-field apparatus (50 × 50 × 50 cm, O’Hara and Corporation Ltd., Tokyo, Japan). Each rat was placed in the center of the open-field apparatus. The center zone was defined as a square, 10 cm away from the wall. The total distance moved, the distance moved in center, and the time spent in center by each animal were recorded for 5 min with a video imaging system (ANY-maze, Stoelting, United States), as previously described (Yoshizaki et al., 2016).

### Golgi-Cox Staining

Golgi-Cox staining was performed to visualize dendritic spine structure in superficial and deep cortical layer neurons using the FD Rapid GolgiStain Kit (FD Neurotechnologies Incorporation, United States). In brief, rats were deeply anesthetized and intracardially perfused with saline. The whole brain was immersed in the A + B solution from the kit for 18 days in the dark at room temperature and then transfer the brain into the C solution from the kit for 72 h in the dark at room temperature. Coronal sections were cut at 100 μm and stained according to FD Rapid GolgiStain’s protocol. The images were captured by VS120 virtual slide microscope (Olympus, Tokyo, Japan).

### Blood–Brain Barrier Permeability Detection

Evans blue (EB) was used to analyze the BBB permeability, as previously described (Naderi et al., 2015). In brief, the rats were anesthetized and injected intravenously with 2% EB (Beijing Yinuokai Technology Corporation Ltd., 3 ml/kg) 1 h before sacrifice. Rats were perfused with saline 24 h after MCAO to remove circulating EB. Brains were harvested and cut into 2 mm slices and the digital photos of EB extravasation were captured. Then, the slice was divided into ischemic and non-ischemic, weighed and stored at –80°C until use. Slices were homogenized in cold PBS and homogenized tissue was soaked up and further homogenized with the same volume of 50% trichloroacetic acid (Sigma-Aldrich). Supernatants were collected after centrifuging at 15,000 rpm for 20 min. Optical density (OD) values of supernatants were measured at 620 nm with a microplate reader (Bio-Rad, Hercules, California, United States). The amount of extravagated EB dye was quantified as microgram per gram brain tissue.

### Terminal Deoxynucleotidyl Transferase-Mediated dUTP Nick-End Labeling (TUNEL) Staining

For TUNEL and CD31 staining in striatum, the brain cryosections (slice thickness 20 μm) were prepared, as previously described (Yang et al., 2012). Briefly, rats were intracardially perfused with saline, followed by 4% PFA perfusion. Then, the brains were immersed in 30% sucrose solution. After dehydration, the brains were embedded in Tissue-TEK OCT Compound (Sakura, United States) and sectioned using freezing microtome (Leica, CM1950). TUNEL staining was performed in accordance with the instructions in the TUNEL Detection Kit (Cat#: PF00009, Proteintech, United States). Mouse antirat CD31 antibody (1:1,000, ab64543, Abcam) and goat antimouse Alexa Fluor 488-labeled secondary antibody (1:1,000; Cat# A11001, Thermo Fisher Scientific) were successively incubated. Then, 4′,6-diamidino-2-phenylindole (DAPI) staining was performed for 20 min and the nucleus was observed under a fluorescence microscope (Olympus BX51, Japan) and the TUNEL/CD31 double-positive cells were counted from ten different fields in each group using Image J software (Maryland, United States).

For cell apoptosis detection, TUNEL detection kit (Cat#: PF00006, Proteintech, United States) was used. The TUNEL^+^ cell percentage was calculated by TUNEL^+^ cell number divided by total cell number per field.

### Cell Culture and Oxygen-Glucose Deprivation/Reoxygenation (OGD/R) Model

The bEnd.3 cell line, immortalized mouse brain endothelial cells, was grown in Dulbecco’s Modified Eagle’s Medium (DMEM) (with 4,500 mg/l D-glucose, 110 mg/l sodium pyruvate, 3,700 mg/l sodium bicarbonate, 584 mg/l L-glutamine, 80 units/ml of penicillin, and 80 μg/ml of streptomycin; KeyGEN BioTECH, Nanjing, China) supplemented with 10% fetal bovine serum (FBS) (Gibco, New York, United States). The bEnd.3 cells were maintained in a humidified incubator at 37°C with 5% CO_2_ and 95% air. In order to simulate an ischemic stroke model *in vitro*, bEnd.3 cells were subjected to OGD/R (Jiang et al., 2000). For OGD/R, the medium was changed to glucose-free DMEM and cultured in an O_2_/CO_2_ (1.2%/5%) trigas incubator. After 9 h, the cells were replaced with the DMEM medium supplemented with 10% FBS and cultured in O_2_/CO_2_ (21/5%) for 24 h (reoxygenation). For cell experiments, the cells were divided into the three groups: control, OGD/R, and OGD/R plus 50 μg/ml Con-exo (labeled as Con-exo).

### Western Blot

The ischemic cerebral cortex was dissected and brain tissue (or serum exosomes) was lysed in radio immunoprecipitation assay (RIPA) buffer with 1X protease and phosphatase inhibitor cocktail. The protein concentration was determined using Bradford protein assay (BCA Protein Assay Kit, Vazyme, China) and normalized for protein content. The lysates were loaded on sodium dodecyl sulfate-polyacrylamide gel electrophoresis (SDS-PAGE) gels and subsequently transferred onto a polyvinylidene difluoride (PVDF) membrane. The PVDF membrane was shaken slowly in the blocking solution (5% milk in Tris-buffered saline with 0.1% Tween 20) at room temperature for 1 h. Then, the membrane was incubated with the diluted primary antibody overnight at 4°C. The secondary antibody was incubated at room temperature for 1 h. The bands were visualized using an enhanced chemiluminescence reagent (KeyGen Biotechnology, China) and analyzed using the Image J software.

Primary antibodies include rabbit anti-CD9 (1:1,000, Proteintech, 20579-1-AP); rabbit anti-TSG101 (1:1,000, Proteintech, 28283-1-AP); rabbit anticleaved caspase-3 (1:1,000, Cell Signaling Technology, 9661); rabbit anti-Bcl-2 (1:1,000, Abcam, ab196495); rabbit anti-Bax (1:1,000, Abcam, ab32503); rabbit anti-ZO-1 (1:1,000, Abcam, ab190085); rabbit anti-MMP-9 (1:1,000, Abcam, ab76003); rabbit anticlaudin-5 (1:1,000, Abcam, ab172968); rabbit anti-LC3B (1:1,000, Sigma-Aldrich, L7543); rabbit anti-SQSTM1/p62 (1:1,000, HuaBio, R1309-8); mouse anti-Akt (1:1,000, Proteintech, 51077-1-AP); rabbit anti-p-Akt (1:1,000, Cell Signaling Technology, S473); and mouse anti-α-tubulin (1:10,000, Proteintech, 66031-1-lg). Secondary antibodies included horseradish peroxidase (HRP)-conjugated antirabbit (1:8,000, Proteintech, SA00001-2) or antimouse immunoglobulin G (IgG) secondary antibodies (1:8,000, Proteintech, SA00001-1).

### Autophagic Morphology Detection

Briefly, cells were harvested after treatment with OGD/R or OGD/R combined with Con-exo, fixed with ice-cold 2.5% glutaraldehyde in PBS (pH 7.3) for 2 h, postfixed in 1% osmium tetroxide, dehydrated in gradient ethanol series (50–100%) and acetone, and embedded in Epon. The sections were stained with 3% lead citrate-uranyl acetate and examined with a TEM (Hitachi, Japan).

### Data Analysis

All the experimental data are shown as means ± SE and analyzed by GraphPad Prism version 8 software (GraphPad Software Incorporation, La Jolla, California, United States). The data for the fluorescence intensity of Dil and spine density were counted by Image J software. The Dil-Con-exo penetration experiment was analyzed by two-way ANOVA. Except that, all the statistical analysis adopted one-way ANOVA; Tukey’s *post hoc* test was used for comparison between each group (α = 0.05); *P* < 0.05 indicated that the difference was statistical significant.

## Results

### Isolation and Characterization of Serum Exosomes

**FIGURE 1:**
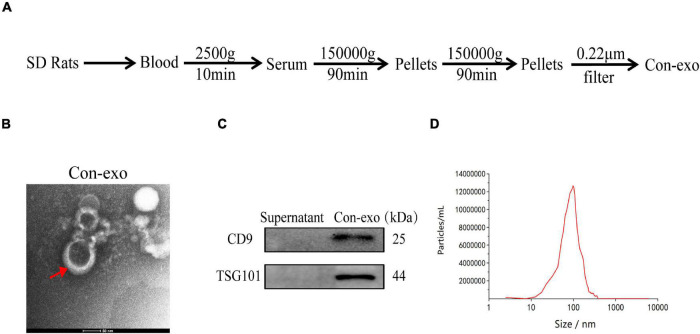
Isolation and characterization of serum exosomes. **(A)** Schematic representation of serum exosomes (Con-exo) isolated from rats. **(B)** Con-exo was photographed by transmission electron microscopy (TEM). Scale bar = 50 nm. **(C)** Representative immunoblotting images of exosomal markers such as CD9 and TSG101 in Con-exo. **(D)** Particle size of Con-exo was measured by nanoparticle tracing analysis.

The serum exosomes (Con-exo) were separated from the blood of healthy rats by gradient centrifugation and ultracentrifugation (Figure 1A). TEM examination indicated Con-exo with typical round morphology (Figure 1B). Exosome-specific surface markers such as CD9 and TSG101 were examined by immunoblotting analysis in exosome lysate and the supernatant from last step of filtration was used as negative control (Figure 1C). The concentration and size distribution of Con-exo were quantified by nanoparticle tracing analysis (NTA). The NTA showed that the particle diameter of the Con-exo ranged from 80 to 200 nm. The distribution width of Con-exo was as following: the X10 was 68.7 ± 10.5 nm, the X50 was 109.6 ± 9.4 nm, and the X90 was 189.8 ± 14.2 nm. The mean diameter of Con-exo was 108.1 ± 15.7 nm (*n* = 3, Figure 1D).

### Serum Exosomes Reduce Infarct Volume and Improve Stroke Outcome

**FIGURE 2:**
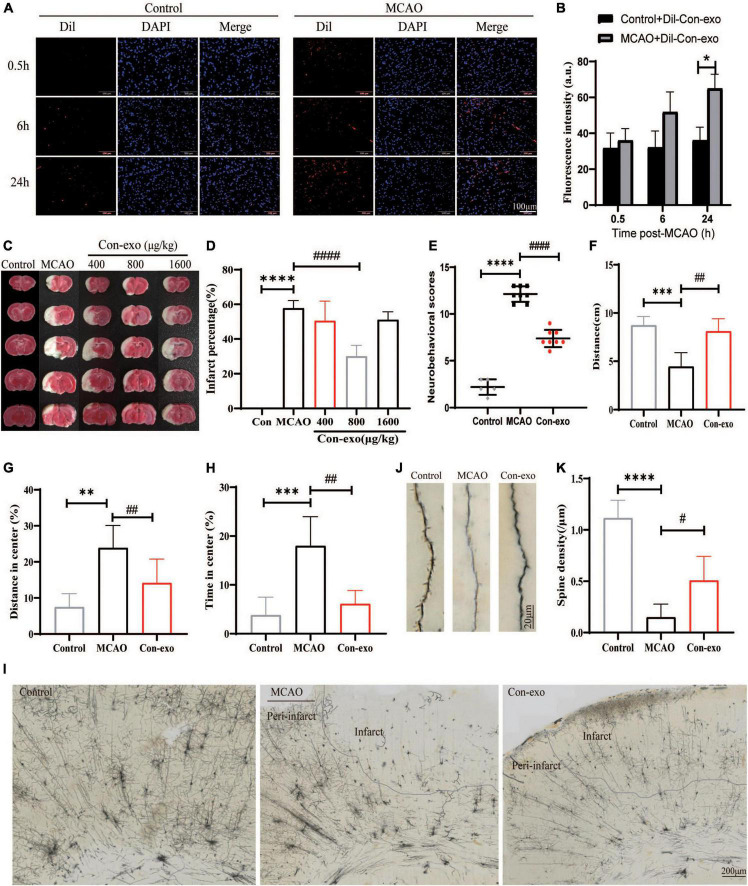
Administration of serum exosomes protect against ischemic damage. **(A,B)** The representative images and statistical analysis of the penetration of Dil-labeled Con-exo accumulating in striatum of control and middle cerebral artery occlusion (MCAO) rats at 0.5, 6, and 24 h. The fluorescence intensity of Dil was counted from five different images of each group. **(C)** Representative 2,3,5-triphenyltetrazolium chloride (TTC)-stained images of phosphate-buffered saline (PBS)-treated (MCAO) and Con-exo (400, 800, and 1,600 μg/kg)-treated brains from ischemic stroke mice (2 h MCAO, 24 h reperfusion). **(D)** Quantitative analysis of the percentage of infarct volume in TTC-stained brains (*n* = 5). **(E)** Neurological deficit of ischemic rats was assessed by neurological severity score in the control, MCAO, and Con-exo groups (*n* = 8). **(F–H)** Motor deficits of ischemic rats were assessed by open field test in the control, MCAO, and Con-exo groups (*n* = 8). Total distance **(F)**, the percentage of distance in center **(G)**, and time **(H)** in center area were detected in each group. **(I)** Representative light microscopic images showing the pattern of Golgi-stained neurons in ipsilateral brains from control, MCAO, and Con-exo rats. **(J)** Representative images of neuronal dendrite and spine segments of each group as indicated. **(K)** Quantitative analysis of the effects of Con-exo on spine density in the peri-infarct cortex. *P < 0.05, **P < 0.01, ***P < 0.001, ****P < 0.0001 vs. control; *^#^P* < 0.05, *^##^P* < 0.01, *^###^P* < 0.001, ^####^*P* < 0.0001 vs. MCAO.

The fluorescence intensity (FI) of Dil in striatum was defined as the ability of Con-exo penetrating BBB. The intensity was quantified in control and MCAO rats at 0.5, 6, and 24 h, respectively. The FI increased over time in MCAO rats. However, compared with the control group, the FI in the MCAO group was strikingly higher at 24 h (compared with MCAO, *P* < 0.05; Figures 2A,B). Then, the rats were subjected to MCAO or sham surgery, with immediate intravenous administration of 400, 800, or 1,600 μg/kg of Con-exo. Rats that received 800 μg/kg Con-exo were found to have significantly reduced infarct volumes compared with PBS-treated animals for 24 h after stroke by TTC staining (compared with MCAO, *P* < 0.0001; Figures 2C,D). Rats that received 800 μg/kg Con-exo treatment at the onset of stroke had significantly improved neurobehavioral scores at 24 h after stroke (*P* < 0.0001) compared with their PBS-treated counterparts (Figure 2E). Con-exo-treated rats had significantly longer total moving distance, less staying time, and shorter moving distance in the center in open field test (compared with MCAO, ***P < 0.001, **P < 0.01, P<0.001; Figures 2F–H). The dendritic spines are the postsynaptic terminals that mediate synaptic transmission and plasticity and effects on the sensorimotor and cognitive deficits of ischemic stroke. We used Golgi-Cox staining to explore the effects of Con-exo on ischemia-induced structural alterations in neuronal dendrites and dendritic spines. The neurons in infarct area lost their dendrites compared with those in peri-infarct area (Figure 2I). However, Con-exo remarkably protected the neuronal spine density against ischemic injury (compared with MCAO, *P* < 0.01; Figures 2J,K).

### Serum Exosomes Reduce the Permeability of the Blood–Brain Barrier Challenged With Ischemic Stimuli

**FIGURE 3:**
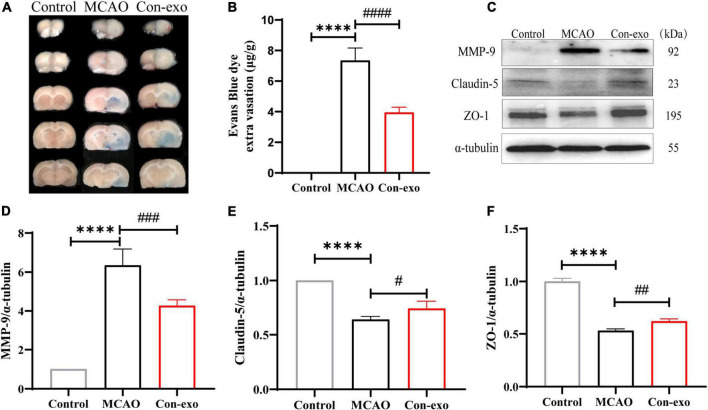
Serum exosomes reduce the blood–brain barrier (BBB) leakage after ischemic stroke. **(A,B)** Representative images and quantitative analysis of the effect of Con-exo on Evans blue (EB) leakage in ischemic brains. **(C–F)** Immunoblotting and statistical analysis of the effect of Con-exo on matrix metalloproteinase-9 (MMP-9) **(D)**, claudin-5 **(E)**, and zonula occludens-1 (ZO-1) **(F)** Expression after ischemic injury. ****P < 0.0001 vs. control, ^#^*P* < 0.05, ^##^*P* < 0.01, ^###^*P* < 0.001, ^####^*P* < 0.0001 vs. MCAO (*n* = 3).

Next, we assessed the permeability of the BBB by measuring EB leakage to explore the neuroprotective mechanism of serum exosome in ischemic brain injury. EB leakage assay revealed that Con-exo obviously reduced the BBB permeability compared with MCAO rats injected with PBS (*P* < 0.0001; Figures 3A,B). To investigate the mechanisms of serum exosomes acting on the BBB modulation, we measured the expression of tight junction proteins, claudin-5 and ZO-1, in ischemic brains. Con-exo remarkably reversed claudin-5 and ZO-1 expression compared with the MCAO group (*P* < 0.05; Figures 3C,E,F). As MMP-9 was the primary proteolytic enzyme, which was reported to degrade claudin-5 and ZO-1 after ischemic stroke, Con-exo significantly inhibited the level of MMP-9 compared with the MCAO group (*P* < 0.01; Figures 3C,D). These data indicated that Con-exo reduced BBB leakage by preserving tight junction proteins in ischemic brains, at least partially owing to the inhibition of MMP-9.

### Serum Exosomes Reduce Endothelial Cell Apoptosis After Ischemic Stroke

**FIGURE 4:**
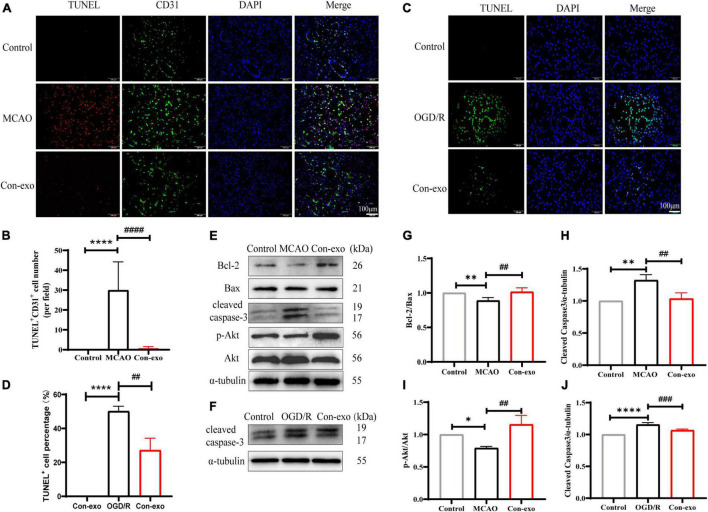
Serum exosomes alleviate apoptosis *in vivo* and *in vitro*. **(A)** The representative images of Con-exo on the endothelial cell apoptosis (TUNEL^+^ CD31^+^ cells) in striatum was evaluated by terminal deoxynucleotidyl transferase (TdT)-mediated dUTP nick-end labeling (TUNEL) staining combined with CD31 immunofluorescence staining. **(B)** The statistical analysis of the effect of Con-exo on TUNEL^+^ CD31^+^ cell number in ischemic striatum. **(C,D)** The representative images and analysis of Con-exo on the apoptosis in oxygen-glucose deprivation/reoxygenation (OGD/R) stimulated bEND.3 cells. **(E,F)** The effect of Con-exo on the expression of cleaved caspase-3, Bcl-2, Bax, p-Akt, and Akt in the ischemic cortex **(E)** and OGD/R stimulated bEND.3 cells **(F)**. **(G–I)** The statistical analysis of cleaved caspase-3 **(G)**, Bcl-2/Bax **(H)**, and p-Akt/Akt **(I)** in the ischemic cortex and cleaved caspase-3 **(J)** in bEND.3 cells challenged by OGD/R. *P < 0.05, **P < 0.01, ****P < 0.0001 vs. control, ^##^*P* < 0.01, ^###^*P* < 0.001, ^####^*P* < 0.0001 vs. MCAO or OGD (*n* = 3).

As the primary cell type of the BBB, cerebrovascular endothelial cell survival is responsible for the BBB integrity. In order to explore the effect of Con-exo on endothelial cell apoptosis after ischemia injury, first, we used TUNEL staining to observe the apoptosis in the ipsilateral cerebrum and simultaneously, endothelial cell marker CD31 was stained for tracing endothelial cell apoptosis. In the MCAO group, the co-localization of TUNEL-positive and CD31-positive cells were obviously increased, compared to the control group. Con-exo treatment significantly reduced the number of TUNEL^+^/CD31^+^ cells compared with the MCAO group (*P* < 0.0001, Figures 4A,B). Compared to the control group, the OGD/R group exhibited significantly increased apoptotic cells. Con-exo treatment reduced the apoptotic cells in bEnd.3 cells (*P* < 0.01, Figures 4C,D). In addition, we analyzed the expression of the apoptosis-related proteins Bcl-2, Bax, and cleaved caspase-3 in the ipsilateral cortex. We found that the ratio of Bcl-2/Bax decreased and cleaved caspase-3 increased in the MCAO group; Con-exo treatment reversed the ratio of Bcl-2/Bax (*P* < 0.01, Figures 4E,G) and the increase of cleaved caspase-3 (*P* < 0.01, Figures 4E,H). We also found that Con-exo could significantly increase the phosphorylation of Akt (Ser473) (*P* < 0.01, Figures 4E,I). Consistently, we obtained the similar results in bEnd.3 cells administrated with Con-exo under OGD/R context (*P* < 0.001, Figures 4F,J). These results demonstrated that Con-exo could reduce endothelial cell apoptosis induced by ischemic injury.

### Serum Exosomes Reduce Autophagy After Ischemic Stroke

**FIGURE 5:**
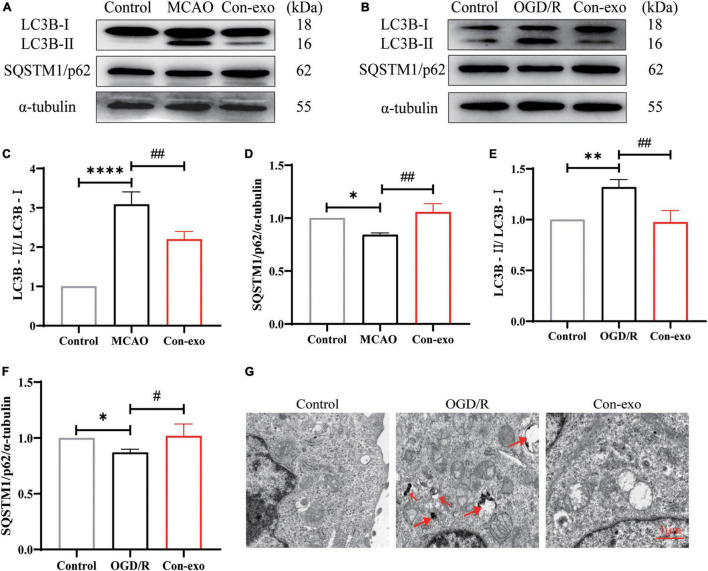
Serum exosomes inhibit autophagy *in vivo* and *in vitro*. **(A,B)** Effect of Con-exo on SQSTM1/p62 and LC3B expression in the ischemic cortex **(A)** and bEnd.3 cells **(B)** as determined by Western blotting analysis. **(C–F)** Statistical analysis of LC3BII/LC3BI **(C,E)** and SQSTM1/p62 **(D,F)** in the ischemic cortex and bEnd.3 cells induced by OGD/R. **(G)** Representative TEM images of autophagic vesicles in bEnd.3 cells induced by OGD/R. *P < 0.05, **P < 0.01, ****P < 0.0001 vs. control, ^#^*P* < 0.05, ^##^*P* < 0.01 vs. MCAO or OGD (*n* = 3).

Recent evidence indicates that autophagy is involved in claudin-5, occludin, and ZO-1 degradation after ischemic stroke and we further examined whether serum exosomes preserved tight junction proteins through autophagy. First, we examined the effect of Con-exo on the expression of autophagic proteins in ischemic brains. Con-exo treatment reduced the ratio of LC3B-II/LC3B-I compared to the MCAO group (*P* < 0.01, Figures 5A,C). During the process of autophagy, SQSTM1/p62 links LC3B-II with ubiquitin moieties on ubiquitinated proteins, which are degraded together. Hence, SQSTM1/p62 expression was reduced in the MCAO group; consistently, SQSTM1/p62 expression was reversed by Con-exo compared with the MCAO group (*P* < 0.01, Figures 5A,D). These findings were further confirmed in bEnd.3 cells (*P* < 0.01, *P* < 0.05, Figures 5B,E,F). TEM revealed that the autolysosomes were remarkably induced after OGD/R, while Con-exo obviously reduced autolysosomes production (Figure 5G).

## Discussion

**FIGURE 6:**
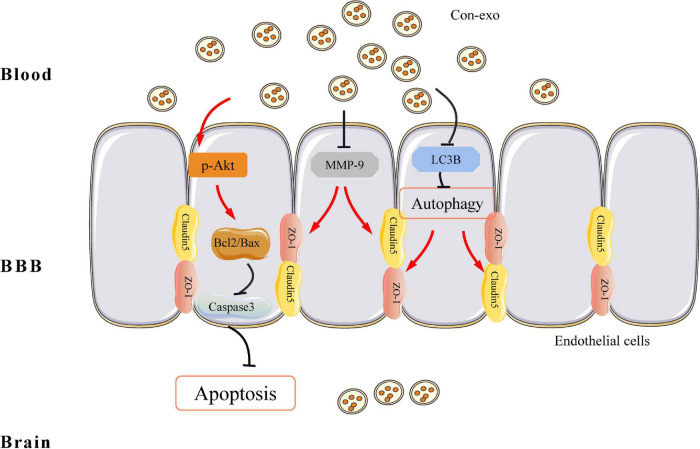
Graphical illustrating of serum exosomes protecting the BBB after ischemia/reperfusion injury. From the antiapoptosis view, serum exosomes enhance the ratio of Bcl2 to Bax and prevent caspase-3 activation via increasing Akt phosphorylation. On top of that, in tight junction’s perspective, serum exosomes reverse the abundance of ZO-1 and claudin-5, while prevent MMP-9 activation. On the other hand, serum exosomes inhibit LC3B-mediated autophagy, which also contributes to the preservation of ZO-1 and claudin-5 under ischemia/reperfusion injury. BBB, blood–brain barrier; Bcl2, B-cell leukemia/lymphoma 2; Bax, Bcl2-associated X protein; Akt, protein kinase B; ZO-1, zonula occludens 1; MMP-9, matrix metalloproteinase-9; LC3B, microtubule-associated protein 1 light chain 3B protein.

During the past years, exosomes have shown positive therapeutic potential in the treatment of cardiovascular and cerebrovascular diseases, which have attracted extensive attention in the field. Among them, animal cells, especially mesenchymal stem cells, macrophages, and exosomes derived from parenchymal cells, have shown good therapeutic prospects. However, obtaining animal cell exosomes require extremely strict cell culture conditions and the potential biosafety issues that may be brought in by the operation procedures and the component in cell culture medium have brought considerable obstacles to the clinical application of exosomes. Recently, both the blood exchange and plasma transfusion have been shown to greatly improve functional recovery after stroke (Ren et al., 2020; Mamtilahun et al., 2021), suggesting that there are active components in the blood that are beneficial to stroke recovery. The clinical promotion of this technology brings great convenience. In this study, we characterized serum exosomes from the blood of healthy rat. This study confirmed that healthy serum exosomes could significantly reduce infarct size and neurological dysfunction in the transient MCAO model of stroke. Our findings illustrated healthy serum exosomes-mediated neuroprotection through protecting the BBB integrity. Finally, we found that serum exosomes protecting the BBB integrity might be attributed to inhibiting endothelial cell apoptosis and autophagy-mediated tight junction protein degradation (Graphical abstract, Figure 6).

Exosomes are rich in parental microRNA, protein, and lipids, hence exosomal microRNAs and proteins are reported as diagnostic or prognostic biomarkers for tumor, stroke, and Parkinson’s disease (Joyce et al., 2016; Li et al., 2018; Wei et al., 2018; Mori et al., 2019; Barbagallo et al., 2020). Recently, the therapeutic potential of cell-derived exosomes and serum or plasma exosomes has attracted great interests. It was reported that blood-derived exosomes from healthy volunteers alleviated impaired motor coordination in 1-Methyl-4-phenyl-1,2,3,6-tetrahydropyridine (MPTP)-treated Parkinson’s disease mice (Sun et al., 2020). Exosomes derived from the serum of patients with myocardial infarction promoted myocardial function recovery (Geng et al., 2020). It was also reported that plasma exosomes protect against cerebral ischemia/reperfusion injury (Chen F. et al., 2015); however, the mechanism of the neuroprotective role of plasma has not been fully elucidated other than reactive oxygen species (ROS) inhibition. We found that serum exosomes had typical round morphology, as previously described (Liu S. et al., 2020). This study demonstrated that 800 μg/kg Con-exo could significantly reduce injury in the ischemic brain. This protection was associated with the decrease in infarct size, neuron dendrite damage, and the BBB destruction. At this moment, we know little about the component in Con-exo; we assume that it may also include the harmful ingredient, which probably diminishes the protection, if overdosed (1,600 μg/kg). The BBB disruption in ischemic stroke occurs dynamically; consistently to previous reports regarding to the short-term protective role on the BBB disruption (Chang et al., 2017; Bao et al., 2018), we chose to observe the protection of Con-exo on 24 h after MCAO. Additionally, we also performed penetration experiments and proved that the penetration of Dil-labeled Con-exo through the BBB strikingly increased at 24 h after MCAO.

The BBB is composed of endothelial cells, pericytes, astrocytes, and tight junction proteins and is surrounded by neurons. Endothelial cell apoptosis also participates in the BBB damage. The primary forms of cell death in ischemic stroke are two major types: a necrotic/necroptotic form and an apoptotic form that are frequently seen in penumbral regions of injury (Dojo Soeandy et al., 2021). Apoptosis can involve either an extrinsic or an intrinsic pathway. Bcl-2 and its family members are required for the intrinsic pathway. Bcl-2, together with Bax, modifies mitochondrial membrane potential and permeability, which releases regulatory proteins that activate cellular caspases, eventually the executor caspase-3 (Wang et al., 2009). We found a striking decline of Bcl-2 and apparent activation of Bax and caspase-3, accompanied by more TUNEL/CD31 double-positive cells in the peri-ischemic cortex after stroke. Although neuronal apoptosis predominantly appeared under ischemic stimuli (Dojo Soeandy et al., 2021), endothelial cell apoptosis was also detectable, which contributed to the BBB disruption as supposed. Phosphatidylinositol 3-kinase/Akt signaling pathway is a classical antiapoptotic pathway, acting through Bcl-2 and caspase-3 (Liu R. et al., 2020). Our results suggested that Con-exo increased the ratio of Bcl-2/Bax, reduced the expression of cleaved caspase-3 in the cortex, and reduced apoptosis in the striatum of cerebral ischemic/reperfusion rats. We also found that Con-exo could significantly increase the phosphorylation of Akt (Ser473). As reported, exosomes containing miR-371b-5p or miR-181b-5p target and degrade phosphatase and tensin homolog (PTEN); once PTEN is inhibited, PI3K/Akt pathway will be activated. Hence, it still deserves further investigation how Con-exo regulates the phosphorylation of Akt (Quan et al., 2017; Lv et al., 2020). Furthermore, Con-exo reduced OGD/R-induced apoptosis in bEnd.3 cells. These above results suggested that Con-exo could reduce apoptosis after cerebral I/R injury by regulating the Akt pathway.

Matrix metalloproteinase-9 activity elevates in the plasma and brains of stroke patients and is identified as a mediator of tight junction disruption associating with brain edema and hemorrhagic transformation (del Zoppo et al., 2007; Sandoval and Witt, 2008; Chen H.S. et al., 2015; Maestrini et al., 2020). MMP-9 increase correlates with an increase in the BBB permeability (Fujimura et al., 1999; Gasche et al., 1999). MMP-9 activation was reported to disrupt the BBB integrity by degrading occludin, ZO-1, and claudin-5, thereby destroying the BBB and leading to cerebral hemorrhage in ischemic brain injury (Yang and Rosenberg, 2011b; Lakhan et al., 2013). Our data showed that Con-exo significantly inhibited the expression of MMP-9; however, it still deserves further investigation whether Con-exo also prevents MMP-9 activation.

Mounting studies have reported that autophagy is involved in neuronal damage and become the therapeutic target for ameliorating ischemic stroke (Feng et al., 2017; Liu et al., 2019; Pei et al., 2019; Wang et al., 2019; Chen W. et al., 2020). On the other hand, autophagy-lysosome pathway plays a key role in the degradation of intracellular damaged organelles and proteins (Huber and Teis, 2016). Autophagy-mediated tight junction protein degradation is critical for maintaining intestinal epithelial barrier and the BBB integrity in ischemic stroke model (Cheng et al., 2018; Luciani et al., 2018; Wong et al., 2019). For example, the BBBs tight junction proteins are involved in arsenic-induced autophagy in developmental mouse cerebral cortex, hippocampus, and cerebellum (Manthari et al., 2018a,b). Zhang et al. (2018) showed that MMP-2/9-mediated extracellular degradation, caveolin-1-mediated intracellular translocation, and autophagy-lysosome-mediated degradation of ZO-1 protein were all involved in the BBB disruption. Autophagy-mediated occludin degradation contributes to the BBB disruption during ischemia, as autophagy inhibition significantly enhanced occludin expression (Wang et al., 2020). Taken together, both the MMP-9 and autophagy contribute to tight junction protein degradation under ischemia/reperfusion injury. Our studies showed that the expression of ZO-1 and claudin-5 reduced in the brain of MCAO rats, intravenous injection of Con-exo (800 μg/kg) remarkably reversed the reduction of ZO-1 and claudin-5, and decreased the expression of MMP-9, LC3B-II/LC3B-I ratio as well as autophagic flux. Exosomes reducing autophagy in stroke mice have been reported (Pei et al., 2019; Chen C.Y. et al., 2020; Fan et al., 2020; Kuang et al., 2020). Consistent with previous studies, our data also revealed the inhibition on autophagy by serum exosomes. Based on these investigations, we concluded that Con-exo protected the BBB integrity that might be at least partially attributed to MMP-9 and autophagy inhibition.

The limitation of this study is that we have not explored the component including small RNAs, proteins, and lipids of the serum exosome and find out the effective ingredient that contributes to the regulation of endothelial cell apoptosis and autophagy under ischemic brain injury. Most importantly, due to the technique limitation, it is impossible to trace the cellular origin of exosome in the serum. Future studies should seek to assess the safety and clinical therapeutic potential of serum exosomes in treating ischemic stroke. Our present evidence brings us the brilliant future in using the serum- or plasma-derived exosomes from healthy donor or the relatives of stroke patients for stroke treatment.

## Data Availability Statement

The original contributions presented in the study are included in the article/Supplementary Material, further inquiries can be directed to the corresponding author/s.

## Ethics Statement

The animal study was reviewed and approved by the Committee on the Use of Live Animals in Teaching and Research of Xuzhou Medical University (License ID: 201907W079).

## Author Contributions

LYH and SHQ designed and coordinated the study. JXS and HC performed the experiments, acquired, and analyzed the data. PPW, QLY, YDZ, and ML helped animal experiments. YLW, LL, and WW interpreted the data. LYH wrote the manuscript. SHQ reviewed the manuscript. All the authors approved the final version of the manuscript.

## Conflict of Interest

The authors declare that the research was conducted in the absence of any commercial or financial relationships that could be construed as a potential conflict of interest.

## Publisher’s Note

All claims expressed in this article are solely those of the authors and do not necessarily represent those of their affiliated organizations, or those of the publisher, the editors and the reviewers. Any product that may be evaluated in this article, or claim that may be made by its manufacturer, is not guaranteed or endorsed by the publisher.

## References

[B1] Abdullahi W. Tripathi D. Ronaldson P. T. (2018). Blood-brain barrier dysfunction in ischemic stroke: targeting tight junctions and transporters for vascular protection. *Am. J. Physiol. Cell Physiol.* 315 C343–C356. 10.1152/ajpcell.00095.2018 29949404 PMC6171039

[B2] Alluri H. Wiggins-Dohlvik K. Davis M. L. Huang J. H. Tharakan B. (2015). Blood-brain barrier dysfunction following traumatic brain injury. *Metab. Brain Dis.* 30 1093–1104. 10.1007/s11011-015-9651-7 25624154

[B3] Armulik A. Genové G. Mäe M. Nisancioglu M. H. Wallgard E. Niaudet C. (2010). Pericytes regulate the blood-brain barrier. *Nature* 468 557–561. 10.1038/nature09522 20944627

[B4] Balkaya M. Kim I.-D. Shakil F. Cho S. (2021). CD36 deficiency reduces chronic BBB dysfunction and scar formation and improves activity, hedonic and memory deficits in ischemic stroke. *J. Cereb. Blood Flow Metab.* 41 486–501. 10.1177/0271678X20924099 32404022 PMC7922745

[B5] Bao Q. Hu P. Xu Y. Cheng T. Wei C. Pan L. (2018). Simultaneous blood-brain barrier crossing and protection for stroke treatment based on edaravone-loaded ceria nanoparticles. *ACS Nano* 12 6794–6805. 10.1021/acsnano.8b01994 29932327

[B6] Barbagallo C. Mostile G. Baglieri G. Giunta F. Luca A. Raciti L. (2020). Specific signatures of serum miRNAs as potential biomarkers to discriminate clinically similar neurodegenerative and vascular-related diseases. *Cell. Mol. Neurobiol.* 40 531–546. 10.1007/s10571-019-00751-y 31691877 PMC11448951

[B7] Bauer A. T. Bürgers H. F. Rabie T. Marti H. H. (2010). Matrix metalloproteinase-9 mediates hypoxia-induced vascular leakage in the brain via tight junction rearrangement. *J. Cereb. Blood Flow Metab.* 30 837–848. 10.1038/jcbfm.2009.248 19997118 PMC2949161

[B8] Bei Y. Chen T. Banciu D. D. Cretoiu D. Xiao J. (2017). Circulating exosomes in cardiovascular diseases. *Adv. Exp. Med. Biol.* 998 255–269. 10.1007/978-981-10-4397-0_17 28936745

[B9] Benjamin E. J. Blaha M. J. Chiuve S. E. Cushman M. Das S. R. Deo R. (2017). Heart disease and stroke statistics-2017 update: a report from the American heart association. *Circulation* 135 e146–e603. 10.1161/CIR.0000000000000485 28122885 PMC5408160

[B10] Blanc L. De Gassart A. Géminard C. Bette-Bobillo P. Vidal M. (2005). Exosome release by reticulocytes–an integral part of the red blood cell differentiation system. *Blood Cells Mol. Dis.* 35 21–26. 10.1016/j.bcmd.2005.04.008 15946868

[B11] Chang J. Mancuso M. R. Maier C. Liang X. Yuki K. Yang L. (2017). Gpr124 is essential for blood-brain barrier integrity in central nervous system disease. *Nat. Med.* 23 450–460. 10.1038/nm.4309 28288111 PMC5559385

[B12] Chen C.Y. Chao Y.-M. Lin H.-F. Chen C.-J. Chen C.-S. Yang J.-L. (2020). miR-195 reduces age-related blood-brain barrier leakage caused by thrombospondin-1-mediated selective autophagy. *Aging Cell* 19:e13236. 10.1111/acel.13236 33029941 PMC7681043

[B13] Chen F. Du Y. Esposito E. Liu Y. Guo S. Wang X. (2015). Effects of focal cerebral ischemia on exosomal versus serum miR126. *Transl. Stroke Res.* 6 478–484. 10.1007/s12975-015-0429-3 26449616

[B14] Chen H.S. Chen X.-M. Feng J.-H. Liu K.-J. Qi S.-H. Shen J.-G. (2015). Peroxynitrite decomposition catalyst reduces delayed thrombolysis-induced hemorrhagic transformation in ischemia-reperfused rat brains. *CNS Neurosci. Ther.* 21 585–590. 10.1111/cns.12406 25996167 PMC6495263

[B15] Chen H. Guan B. Wang B. Pu H. Bai X. Chen X. (2019). Glycyrrhizin prevents hemorrhagic transformation and improves neurological outcome in ischemic stroke with delayed thrombolysis through targeting peroxynitrite-mediated HMGB1 signaling. *Transl. Stroke Res* 11 967–982. 10.1007/s12975-019-00772-1 31872339

[B16] Chen J. Chopp M. (2018). Exosome therapy for stroke. *Stroke* 49 1083–1090. 10.1161/STROKEAHA.117.018292 29669873 PMC6028936

[B17] Chen J. Sanberg P. R. Li Y. Wang L. Lu M. Willing A. E. (2001). Intravenous administration of human umbilical cord blood reduces behavioral deficits after stroke in rats. *Stroke* 32 2682–2688. 10.1161/hs1101.098367 11692034

[B18] Chen W. Wang H. Zhu Z. Feng J. Chen L. (2020). Exosome-shuttled circSHOC2 from IPASs regulates neuronal autophagy and ameliorates ischemic brain injury via the miR-7670-3p/SIRT1 axis. *Mol. Ther. Nucleic Acids* 22 657–672. 10.1016/j.omtn.2020.09.027 33230464 PMC7581834

[B19] Cheng S. Ma X. Geng S. Jiang X. Li Y. Hu L. (2018). Fecal microbiota transplantation beneficially regulates intestinal mucosal autophagy and alleviates gut barrier injury. *mSystems* 3:e00137-18. 10.1128/mSystems.00137-18 30320222 PMC6178585

[B20] Colombo M. Raposo G. Théry C. (2014). Biogenesis, secretion, and intercellular interactions of exosomes and other extracellular vesicles. *Annu. Rev. Cell Dev. Biol.* 30 255–289. 10.1146/annurev-cellbio-101512-122326 25288114

[B21] del Zoppo G. J. Milner R. Mabuchi T. Hung S. Wang X. Berg G. I. (2007). Microglial activation and matrix protease generation during focal cerebral ischemia. *Stroke* 38 646–651. 10.1161/01.STR.0000254477.34231.cb 17261708

[B22] Doeppner T. R. Herz J. Görgens A. Schlechter J. Ludwig A.-K. Radtke S. (2015). Extracellular vesicles improve post-stroke neuroregeneration and prevent postischemic immunosuppression. *Stem Cells Transl. Med.* 4 1131–1143. 10.5966/sctm.2015-0078 26339036 PMC4572905

[B23] Dojo Soeandy C. Elia A. J. Cao Y. Rodgers C. Huang S. Elia A. C. (2021). Necroptotic-apoptotic regulation in an endothelin-1 model of cerebral ischemia. *Cell. Mol. Neurobiol.* 41 1727–1742. 10.1007/s10571-020-00942-y 32844322 PMC11444014

[B24] ElAli A. Doeppner T. R. Zechariah A. Hermann D. M. (2011). Increased blood-brain barrier permeability and brain edema after focal cerebral ischemia induced by hyperlipidemia: role of lipid peroxidation and calpain-1/2, matrix metalloproteinase-2/9, and RhoA overactivation. *Stroke* 42 3238–3244. 10.1161/STROKEAHA.111.615559 21836084

[B25] Elashiry M. Elsayed R. Elashiry M. M. Rashid M. H. Ara R. Arbab A. S. (2021). Proteomic characterization. biodistribution, and functional studies of immune-therapeutic exosomes: implications for inflammatory lung diseases. *Front. Immunol.* 12:636222. 10.3389/fimmu.2021.636222 33841418 PMC8027247

[B26] Fan Y. Li Y. Huang S. Xu H. Li H. Liu B. (2020). Resveratrol-primed exosomes strongly promote the recovery of motor function in SCI rats by activating autophagy and inhibiting apoptosis via the PI3K signaling pathway. *Neurosci. Lett.* 736:135262. 10.1016/j.neulet.2020.135262 32682847

[B27] Feng D. Wang B. Wang L. Abraham N. Tao K. Huang L. (2017). Pre-ischemia melatonin treatment alleviated acute neuronal injury after ischemic stroke by inhibiting endoplasmic reticulum stress-dependent autophagy via PERK and IRE1 signalings. *J. Pineal Res.* 62:e12395. 10.1111/jpi.12395 28178380

[B28] Fujimura M. Gasche Y. Morita-Fujimura Y. Massengale J. Kawase M. Chan P. H. (1999). Early appearance of activated matrix metalloproteinase-9 and blood-brain barrier disruption in mice after focal cerebral ischemia and reperfusion. *Brain Res.* 842 92–100. 10.1016/s0006-8993(99)01843-0 10526099

[B29] Gao Y. Jiang B. Sun H. Ru X. Sun D. Wang L. (2018). The burden of stroke in China: results from a nationwide population-based epidemiological survey. *PLoS One* 13:e0208398. 10.1371/journal.pone.0208398 30521583 PMC6283556

[B30] Gasche Y. Fujimura M. Morita-Fujimura Y. Copin J. C. Kawase M. Massengale J. (1999). Early appearance of activated matrix metalloproteinase-9 after focal cerebral ischemia in mice: a possible role in blood-brain barrier dysfunction. *J. Cereb. Blood Flow Metab.* 19 1020–1028. 10.1097/00004647-199909000-00010 10478654

[B31] Geng T. Song Z.-Y. Xing J.-X. Wang B.-X. Dai S.-P. Xu Z.-S. (2020). Exosome derived from coronary serum of patients with myocardial infarction promotes angiogenesis through the miRNA-143/IGF-IR pathway. *Int. J. Nanomedicine* 15 2647–2658. 10.2147/IJN.S242908 32368046 PMC7183550

[B32] Gong J. Sun F. Li Y. Zhou X. Duan Z. Duan F. (2015). *Momordica charantia* polysaccharides could protect against cerebral ischemia/reperfusion injury through inhibiting oxidative stress mediated c-Jun N-terminal kinase 3 signaling pathway. *Neuropharmacology* 91 123–134. 10.1016/j.neuropharm.2014.11.020 25510970

[B33] Hu J.-Z. Wang X.-K. Cao Y. Li D.-Z. Wu T.-D. Zhang T. (2017). Tetramethylpyrazine facilitates functional recovery after spinal cord injury by inhibiting MMP2, MMP9, and vascular endothelial cell apoptosis. *Curr. Neurovasc. Res.* 14 110–116. 10.2174/1567202614666170313114115 28294065

[B34] Huber L. A. Teis D. (2016). Lysosomal signaling in control of degradation pathways. *Curr. Opin. Cell Biol.* 39 8–14. 10.1016/j.ceb.2016.01.006 26827287

[B35] Jiang Q. Gu Z. Zhang G. Jing G. (2000). N-methyl-D-aspartate receptor activation results in regulation of extracellular signal-regulated kinases by protein kinases and phosphatases in glutamate-induced neuronal apototic-like death. *Brain Res.* 887 285–292. 10.1016/s0006-8993(00)03003-1 11134617

[B36] Jiang X. Andjelkovic A. V. Zhu L. Yang T. Bennett M. V. L. Chen J. (2018). Blood-brain barrier dysfunction and recovery after ischemic stroke. *Prog. Neurobiol.* 16 144–171. 10.1016/j.pneurobio.2017.10.001 28987927 PMC5886838

[B37] Jiang Y. He R. Shi Y. Liang J. Zhao L. (2020). Plasma exosomes protect against cerebral ischemia/reperfusion injury via exosomal HSP70 mediated suppression of ROS. *Life Sci* 256:117987. 10.1016/j.lfs.2020.117987 32569778

[B38] Joyce D. P. Kerin M. J. Dwyer R. M. (2016). Exosome-encapsulated microRNAs as circulating biomarkers for breast cancer. *Int. J. Cancer* 139 1443–1448. 10.1002/ijc.30179 27170104

[B39] Kang J.-Y. Park H. Kim H. Mun D. Park H. Yun N. (2019). Human peripheral blood−derived exosomes for microRNA delivery. *Int. J. Mol. Med.* 43 2319–2328. 10.3892/ijmm.2019.4150 30942393 PMC6488179

[B40] Kim K.-A. Kim D. Kim J.-H. Shin Y.-J. Kim E.-S. Akram M. (2020). Autophagy-mediated occludin degradation contributes to blood-brain barrier disruption during ischemia in bEnd.3 brain endothelial cells and rat ischemic stroke models. *Fluids Barriers CNS* 17:21. 10.1186/s12987-020-00182-8 32169114 PMC7071658

[B41] Kuang Y. Zheng X. Zhang L. Ai X. Venkataramani V. Kilic E. (2020). Adipose-derived mesenchymal stem cells reduce autophagy in stroke mice by extracellular vesicle transfer of miR-25. *J. Extracell. Vesicles* 10:e12024. 10.1002/jev2.12024 33304476 PMC7710129

[B42] Kumari R. Willing L. B. Patel S. D. Baskerville K. A. Simpson I. A. (2011). Increased cerebral matrix metalloprotease-9 activity is associated with compromised recovery in the diabetic db/db mouse following a stroke. *J. Neurochem.* 119 1029–1040. 10.1111/j.1471-4159.2011.07487.x 21923664 PMC3217107

[B43] Lakhan S. E. Kirchgessner A. Tepper D. Leonard A. (2013). Matrix metalloproteinases and blood-brain barrier disruption in acute ischemic stroke. *Front. Neurol.* 4:32. 10.3389/fneur.2013.00032 23565108 PMC3615191

[B44] Li D.-B. Liu J.-L. Wang W. Luo X.-M. Zhou X. Li J.-P. (2018). Plasma exosomal miRNA-122-5p and miR-300-3p as potential markers for transient ischaemic attack in rats. *Front. Aging Neurosci.* 10:24. 10.3389/fnagi.2018.00024 29467645 PMC5808157

[B45] Li Y. Ren C. Li H. Jiang F. Wang L. Xia C. (2019). Role of exosomes induced by remote ischemic preconditioning in neuroprotection against cerebral ischemia. *Neuroreport* 30 834–841. 10.1097/WNR.0000000000001280 31283710

[B46] Liu J. Jin X. Liu K. J. Liu W. (2012). Matrix metalloproteinase-2-mediated occludin degradation and caveolin-1-mediated claudin-5 redistribution contribute to blood-brain barrier damage in early ischemic stroke stage. *J. Neurosci.* 32 3044–3057. 10.1523/JNEUROSCI.6409-11.2012 22378877 PMC3339570

[B47] Liu R. Chen Y. Liu G. Li C. Song Y. Cao Z. (2020). PI3K/AKT pathway as a key link modulates the multidrug resistance of cancers. *Cell Death Dis.* 11:797. 10.1038/s41419-020-02998-6 32973135 PMC7515865

[B48] Liu S. Agalliu D. Yu C. Fisher M. (2012). The role of pericytes in blood-brain barrier function and stroke. *Curr. Pharm. Des.* 18 3653–3662. 10.2174/138161212802002706 22574979

[B49] Liu S. Lin Z. Zheng Z. Rao W. Lin Y. Chen H. (2020). Serum exosomal microRNA-766-3p expression is associated with poor prognosis of esophageal squamous cell carcinoma. *Cancer Sci.* 111 3881–3892. 10.1111/cas.14550 32589328 PMC7540979

[B50] Liu Y. Xue X. Zhang H. Che X. Luo J. Wang P. (2019). Neuronal-targeted TFEB rescues dysfunction of the autophagy-lysosomal pathway and alleviates ischemic injury in permanent cerebral ischemia. *Autophagy* 15 493–509. 10.1080/15548627.2018.1531196 30304977 PMC6351122

[B51] Longa E. Z. Weinstein P. R. Carlson S. Cummins R. (1989). Reversible middle cerebral artery occlusion without craniectomy in rats. *Stroke* 20 84–91. 10.1161/01.str.20.1.84 2643202

[B52] Luciani A. Festa B. P. Chen Z. Devuyst O. (2018). Defective autophagy degradation and abnormal tight junction-associated signaling drive epithelial dysfunction in cystinosis. *Autophagy* 14 1157–1159. 10.1080/15548627.2018.1446625 29806776 PMC6103718

[B53] Lv P.-Y. Gao P.-F. Tian G.-J. Yang Y.-Y. Mo F.-F. Wang Z.-H. (2020). Osteocyte-derived exosomes induced by mechanical strain promote human periodontal ligament stem cell proliferation and osteogenic differentiation via the miR-181b-5p/PTEN/AKT signaling pathway. *Stem Cell Res. Ther.* 11:295. 10.1186/s13287-020-01815-3 32680565 PMC7367226

[B54] Maestrini I. Tagzirt M. Gautier S. Dupont A. Mendyk A.-M. Susen S. (2020). Analysis of the association of MPO and MMP-9 with stroke severity and outcome: cohort study. *Neurology* 95 e97–e108. 10.1212/WNL.0000000000009179 32111692

[B55] Mamtilahun M. Jiang L. Song Y. Shi X. Liu C. Jiang Y. (2021). Plasma from healthy donors protects blood-brain barrier integrity via FGF21 and improves the recovery in a mouse model of cerebral ischaemia. *Stroke Vasc. Neurol.* 6 561–571. 10.1136/svn-2020-000774 33785536 PMC8717795

[B56] Manthari R. K. Tikka C. Ommati M. M. Niu R. Sun Z. Wang J. (2018a). Arsenic-induced autophagy in the developing mouse cerebellum: involvement of the blood-brain barrier’s tight-junction proteins and the PI3K-Akt-mTOR signaling pathway. *J. Agric. Food Chem.* 66 8602–8614. 10.1021/acs.jafc.8b02654 30032600

[B57] Manthari R. K. Tikka C. Ommati M. M. Niu R. Sun Z. Wang J. (2018b). Arsenic induces autophagy in developmental mouse cerebral cortex and hippocampus by inhibiting PI3K/Akt/mTOR signaling pathway: involvement of blood-brain barrier’s tight junction proteins. *Arch. Toxicol.* 92 3255–3275. 10.1007/s00204-018-2304-y 30225639

[B58] Mori M. A. Ludwig R. G. Garcia-Martin R. Brandão B. B. Kahn C. R. (2019). Extracellular miRNAs: from biomarkers to mediators of physiology and disease. *Cell Metab.* 30 656–673. 10.1016/j.cmet.2019.07.011 31447320 PMC6774861

[B59] Mracsko E. Veltkamp R. (2014). Neuroinflammation after intracerebral hemorrhage. *Front. Cell. Neurosci.* 8:388. 10.3389/fncel.2014.00388 25477782 PMC4238323

[B60] Naderi V. Khaksari M. Abbasi R. Maghool F. (2015). Estrogen provides neuroprotection against brain edema and blood brain barrier disruption through both estrogen receptors α and β following traumatic brain injury. *Iran. J. Basic Med. Sci.* 18 138–144. 25810887 PMC4366724

[B61] Naito M. G. Xu D. Amin P. Lee J. Wang H. Li W. (2020). Sequential activation of necroptosis and apoptosis cooperates to mediate vascular and neural pathology in stroke. *Proc. Natl. Acad. Sci. U.S.A.* 117 4959–4970. 10.1073/pnas.1916427117 32071228 PMC7060720

[B62] Nozohouri S. Vaidya B. Abbruscato T. J. (2020). Exosomes in ischemic stroke. *Curr. Pharm. Des.* 26 5533–5545. 10.2174/1381612826666200614180253 32534564

[B63] Page S. Patel R. Raut S. Al-Ahmad A. (2020). Neurological diseases at the blood-brain barrier: stemming new scientific paradigms using patient-derived induced pluripotent cells. *Biochim. Biophys. Acta Mol. Basis Dis.* 1866:165358. 10.1016/j.bbadis.2018.12.009 30593893

[B64] Pei X. Li Y. Zhu L. Zhou Z. (2019). Astrocyte-derived exosomes suppress autophagy and ameliorate neuronal damage in experimental ischemic stroke. *Exp. Cell Res.* 382:111474. 10.1016/j.yexcr.2019.06.019 31229506

[B65] Qi Z. Liang J. Pan R. Dong W. Shen J. Yang Y. (2016). Zinc contributes to acute cerebral ischemia-induced blood-brain barrier disruption. *Neurobiol. Dis.* 95 12–21. 10.1016/j.nbd.2016.07.003 27388935

[B66] Quan Y. Wang Z. Gong L. Peng X. Richard M. A. Zhang J. (2017). Exosome miR-371b-5p promotes proliferation of lung alveolar progenitor type II cells by using PTEN to orchestrate the PI3K/Akt signaling. *Stem Cell Res. Ther.* 8:138. 10.1186/s13287-017-0586-2 28595637 PMC5465462

[B67] Reeson P. Tennant K. A. Gerrow K. Wang J. Weiser Novak S. Thompson K. (2015). Delayed inhibition of VEGF signaling after stroke attenuates blood-brain barrier breakdown and improves functional recovery in a comorbidity-dependent manner. *J. Neurosci.* 35 5128–5143. 10.1523/JNEUROSCI.2810-14.2015 25834040 PMC6705411

[B68] Ren X. Hu H. Farooqi I. Simpkins J. W. (2020). Blood substitution therapy rescues the brain of mice from ischemic damage. *Nat. Commun.* 11:4078. 10.1038/s41467-020-17930-x 32843630 PMC7447645

[B69] Reuter B. Rodemer C. Grudzenski S. Meairs S. Bugert P. Hennerici M. G. (2015). Effect of simvastatin on MMPs and TIMPs in human brain endothelial cells and experimental stroke. *Transl. Stroke Res.* 6 156–159. 10.1007/s12975-014-0381-7 25476155

[B70] Robbins P. D. Dorronsoro A. Booker C. N. (2016). Regulation of chronic inflammatory and immune processes by extracellular vesicles. *J. Clin. Invest.* 126 1173–1180. 10.1172/JCI81131 27035808 PMC4811148

[B71] Sacks D. Baxter B. Campbell B. C. V. Carpenter J. S. Cognard C. Dippel D. (2018). Multisociety consensus quality improvement revised consensus statement for endovascular therapy of acute ischemic stroke. *Int. J. Stroke* 13 612–632. 10.1177/1747493018778713 29786478

[B72] Sandoval K. E. Witt K. A. (2008). Blood-brain barrier tight junction permeability and ischemic stroke. *Neurobiol. Dis.* 32 200–219. 10.1016/j.nbd.2008.08.005 18790057

[B73] Song D. Jiang X. Liu Y. Sun Y. Cao S. Zhang Z. (2018). Asiaticoside attenuates cell growth inhibition and apoptosis induced by Aβ(1-42) via inhibiting the TLR4/NF-κB signaling pathway in human brain microvascular endothelial cells. *Front. Pharmacol.* 9:28. 10.3389/fphar.2018.00028 29441018 PMC5797575

[B74] Song Y. Li Z. He T. Qu M. Jiang L. Li W. (2019). M2 microglia-derived exosomes protect the mouse brain from ischemia-reperfusion injury via exosomal miR-124. *Theranostics* 9 2910–2923. 10.7150/thno.30879 31244932 PMC6568171

[B75] Sun T. Ding Z.-X. Luo X. Liu Q.-S. Cheng Y. (2020). Blood exosomes have neuroprotective effects in a mouse model of Parkinson’s disease. *Oxid. Med. Cell. Longev.* 2020:3807476. 10.1155/2020/3807476 33294121 PMC7714585

[B76] Thomsen M. S. Routhe L. J. Moos T. (2017). The vascular basement membrane in the healthy and pathological brain. *J. Cereb. Blood Flow Metab.* 37 3300–3317. 10.1177/0271678X17722436 28753105 PMC5624399

[B77] Turner R. J. Sharp F. R. (2016). Implications of MMP9 for blood brain barrier disruption and hemorrhagic transformation following ischemic stroke. *Front. Cell. Neurosci.* 10:56. 10.3389/fncel.2016.00056 26973468 PMC4777722

[B78] Wang J. Qi L. Zheng S. Wu T. (2009). Curcumin induces apoptosis through the mitochondria-mediated apoptotic pathway in HT-29 cells. *J. Zhejiang Univ. Sci. B* 10 93–102. 10.1631/jzus.B0820238 19235267 PMC2644749

[B79] Wang M. Liang X. Cheng M. Yang L. Liu H. Wang X. (2019). Homocysteine enhances neural stem cell autophagy in in vivo and in vitro model of ischemic stroke. *Cell Death Dis.* 10:561. 10.1038/s41419-019-1798-4 31332165 PMC6646339

[B80] Wang S. Xu J. Xi J. Grothusen J. R. Liu R. (2020). Autophagy inhibition preserves tight junction of human cerebral microvascular endothelium under oxygen glucose deprivation. *Curr. Neurovasc. Res.* 17 644–651. 10.2174/1567202617999201103200705 33155911

[B81] Wang Z. Leng Y. Tsai L.-K. Leeds P. Chuang D.-M. (2011). Valproic acid attenuates blood-brain barrier disruption in a rat model of transient focal cerebral ischemia: the roles of HDAC and MMP-9 inhibition. *J. Cereb. Blood Flow Metab.* 31 52–57. 10.1038/jcbfm.2010.195 20978517 PMC3049473

[B82] Wei H. Xu Y. Xu W. Zhou Q. Chen Q. Yang M. (2018). Serum Exosomal miR-223 Serves as a potential diagnostic and prognostic biomarker for dementia. *Neuroscience* 379 167–176. 10.1016/j.neuroscience.2018.03.016 29559383

[B83] Wong M. Ganapathy A. S. Suchanec E. Laidler L. Ma T. Nighot P. (2019). Intestinal epithelial tight junction barrier regulation by autophagy-related protein ATG6/beclin 1. *Am. J. Physiol. Cell Physiol.* 316 C753–C765. 10.1152/ajpcell.00246.2018 30892937 PMC6580157

[B84] Wu F. Xu K. Xu K. Teng C. Zhang M. Xia L. (2020). Dl-3n-butylphthalide improves traumatic brain injury recovery via inhibiting autophagy-induced blood-brain barrier disruption and cell apoptosis. *J. Cell. Mol. Med.* 24 1220–1232. 10.1111/jcmm.14691 31840938 PMC6991645

[B85] Yang G. Wang Y. Zeng Y. Gao G. F. Liang X. Zhou M. (2013). Rapid health transition in China, 1990-2010: findings from the Global Burden of Disease Study 2010. *Lancet* 381 1987–2015. 10.1016/S0140-6736(13)61097-1 23746901 PMC7159289

[B86] Yang H. Huang L.-Y. Zeng D.-Y. Huang E.-W. Liang S.-J. Tang Y.-B. (2012). Decrease of intracellular chloride concentration promotes endothelial cell inflammation by activating nuclear factor-kappaB pathway. *Hypertension* 60 1287–1293. 10.1161/HYPERTENSIONAHA.112.198648 23006728

[B87] Yang Y. Rosenberg G. A. (2011a). Blood-brain barrier breakdown in acute and chronic cerebrovascular disease. *Stroke* 42 3323–3328. 10.1161/STROKEAHA.110.608257 21940972 PMC3584169

[B88] Yang Y. Rosenberg G. A. (2011b). MMP-mediated disruption of claudin-5 in the blood-brain barrier of rat brain after cerebral ischemia. *Methods Mol. Biol.* 762 333–345. 10.1007/978-1-61779-185-7_24 21717368 PMC4950933

[B89] Yang Z. Lin P. Chen B. Zhang X. Xiao W. Wu S. (2021). Autophagy alleviates hypoxia-induced blood-brain barrier injury via regulation of CLDN5 (claudin 5). *Autophagy* 17 3048–3067. 10.1080/15548627.2020.1851897 33280500 PMC8526012

[B90] Yong Y.-X. Yang H. Lian J. Xu X.-W. Han K. Hu M.-Y. (2019). Up-regulated microRNA-199b-3p represses the apoptosis of cerebral microvascular endothelial cells in ischemic stroke through down-regulation of MAPK/ERK/EGR1 axis. *Cell Cycle* 18 1868–1881. 10.1080/15384101.2019.1632133 31204565 PMC6681782

[B91] Yoshizaki K. Furuse T. Kimura R. Tucci V. Kaneda H. Wakana S. (2016). Paternal aging affects behavior in Pax6 mutant mice: a gene/environment interaction in understanding neurodevelopmental disorders. *PLoS One* 11:e0166665. 10.1371/journal.pone.0166665 27855195 PMC5113965

[B92] Zhang G. Zhu Z. Wang H. Yu Y. Chen W. Waqas A. (2020). Exosomes derived from human neural stem cells stimulated by interferon gamma improve therapeutic ability in ischemic stroke model. *J. Adv. Res.* 24 435–445. 10.1016/j.jare.2020.05.017 32551140 PMC7289755

[B93] Zhang S. An Q. Wang T. Gao S. Zhou G. (2018). Autophagy- and MMP-2/9-mediated reduction and redistribution of ZO-1 contribute to hyperglycemia-increased blood-brain barrier permeability during early reperfusion in stroke. *Neuroscience* 377 126–137. 10.1016/j.neuroscience.2018.02.035 29524637

