# Supplementary material for: Healthy Serum-Derived Exosomes Improve Neurological Outcomes and Protect Blood–Brain Barrier by Inhibiting Endothelial Cell Apoptosis and Reversing Autophagy-Mediated Tight Junction Protein Reduction in Rat Stroke Model
Source: Front Cell Neurosci. 2022 Mar 3;16:841544. doi: 10.3389/fncel.2022.841544 (PMC8927286; doi:10.3389/fncel.2022.841544)
Supplement: Supplementary file 1 [file Table_1.docx]

**Supplementary file 1. mNSS score**

| **Motor tests** |  |
| --- | --- |
| ***Raising rat by tail*** | 3 |
| Flexion of forelimb | 1 |
| Flexion of hindlimb | 1 |
| Head moved 10° to vertical axis within 30 s | 1 |
| ***Placing rat on floor (normal0; maximum3)*** | 3 |
| Normal walk | 0 |
| Inability to walk straight | 1 |
| Circling toward the paretic side | 2 |
| Falls down to the paretic side | 3 |
| **Sensory tests** | 2 |
| Placing test (visual and tactile test) | 1 |
| Proprioceptive test (deep sensation, pushing paw against table edge to stimulate limb muscles) | 1 |
| **Beam balance tests (normal0; maximum6)** | 6 |
| Balances with steady posture 0 | 0 |
| Grasps side of the beam | 1 |
| Hugs beam and 1 limb falls down from the beam | 2 |
| Hugs beam and 2 limbs fall down from the beam, or spins on beam (60 s) | 3 |
| Attempts to balance on beam but falls off (40 s) | 4 |
| Attempts to balance on beam but falls off (20 s) | 5 |
| Falls off; no attempt to balance or hang on to beam (20 s) | 6 |
| **Reflex absence and abnormal movements** | 4 |
| Pinna reflex (head shake when auditory meatus is touched) | 1 |
| Corneal reflex (eye blink when cornea is lightly touched with cotton) | 1 |
| Startle reflex (motor response to a brief noise from snapping a clipboard paper) | 1 |
| Seizures, myoclonus, myodystony | 1 |
| **Maximum points** | 18 |
